# How neuronal correlations affect the LFP signal?

**DOI:** 10.1186/1471-2202-16-S1-P60

**Published:** 2015-12-18

**Authors:** Bartosz Telenczuk, Alain Destexhe

**Affiliations:** 1 Unité de Neurosciences, Information & Complexité, Centre National de la Recherche Scientifique, 91198 Gif-sur-Yvette, France

## 

Local field potential (LFP) remains a prime source of information about the activity of the living brain. It allows to monitor the ensemble activity of a large neuronal population at high temporal resolution. Nevertheless, it is notoriously hard to analyze and interpret in terms of underlying neuronal processes. Although the LFP is a population phenomenon, most systematic efforts to understand it concentrated on single or multiple independent sources. Over past few years it became clear that the activities of individual neurons in vivo are strongly correlated. Such correlations could be introduced internally by direct coupling between the neurons and by shared inputs or externally by incoming stimuli. To study the effect of these correlations on the LFP, we introduce a simple and analytically tractable LFP model. This model consists of a population of correlated Poisson neurons. Spikes of these neurons contribute a fixed amount to the LFP signal, which is modeled by convolution of the concatenated spike trains with a temporal waveform describing the contribution (a LFP kernel). We show that the cross-correlations between neurons in the population can account for multiple, seemingly unrelated experimental observations:

1. *Multi-unit activity correlates best with high-gamma (60 -- 100 Hz) band of the LFP *[1]. We show that in a population of uncorrelated neurons, the LFP power is proportional to firing rate, but it scales with its square when neurons fire correlated spikes. The effect of the correlations on the LFP is frequency-dependent -- for realistic values of spike jitter the LFP spectrum peaks in high-gamma-range frequencies explaining their sensitivity to modulations of multi-unit activity.

2. *Single neuron contribution to LFP estimated from experimental data is non-causal and spreads over large distances from the n*euron [2]. We show that the estimate of the spike contribution to the LFP, the spike-triggered LFP average (st-LFP), sums the direct contribution of the trigger neuron and contributions from all correlated neurons. Since the correlations extend in time (due to spike jitter) and in space (due to connectivity patterns), they lead to spatio-temporal smoothing of the direct (causal and local) contribution.

3. *A single spike can initiate a propagating wave in the local neighborhood of a neuron *[3]. We found that the cross-correlogram between pairs of cortical neurons manifest characteristic widening with distance [2]. We show that in our model this widening corresponds to increasingly strong temporal smoothing, which delays the st-LFP peak and produces an apparent propagation of the spike-evoked field.

4. *st-LFP resembles in magnitude and waveform the cross-correlation between LFP and membrane potential (LFP-Vm) *[4]. Both st-LFP and LFP-Vm are strongly affected by the correlations between neurons. In our model the st-LFP and LFP-Vm are dissimilar in absence of correlations and approach each other when correlations gradually increase.

We conclude that the specifics of neuronal correlations can affect the LFP signals as much as the medium properties or spatial distribution of the sources. Since the correlations in vivo are often strong and are modulated by external stimuli and the brain state, such correlations may be crucial for interpreting the LFP data.
